# Variation in helper effort among cooperatively breeding bird species is consistent with Hamilton's Rule

**DOI:** 10.1038/ncomms12663

**Published:** 2016-08-24

**Authors:** Jonathan P. Green, Robert P. Freckleton, Ben J. Hatchwell

**Affiliations:** 1 Department of Animal and Plant Sciences, University of Sheffield, Sheffield S10 2TN, UK

## Abstract

Investment by helpers in cooperative breeding systems is extremely variable among species, but this variation is currently unexplained. Inclusive fitness theory predicts that, all else being equal, cooperative investment should correlate positively with the relatedness of helpers to the recipients of their care. We test this prediction in a comparative analysis of helper investment in 36 cooperatively breeding bird species. We show that species-specific helper contributions to cooperative brood care increase as the mean relatedness between helpers and recipients increases. Helper contributions are also related to the sex ratio of helpers, but neither group size nor the proportion of nests with helpers influence helper effort. Our findings support the hypothesis that variation in helping behaviour among cooperatively breeding birds is consistent with Hamilton's rule, indicating a key role for kin selection in the evolution of cooperative investment in social birds.

## Abstract (web summary)

Non-parental helpers contribute to offspring care in many species; however, the amount of care provided varies considerably across species. Here, Green *et al*. perform a phylogenetic comparative analysis of helping behavior in 36 cooperatively-breeding bird species and find that helper effort increases with relatedness to the recipient of care.

## Graphical abstract

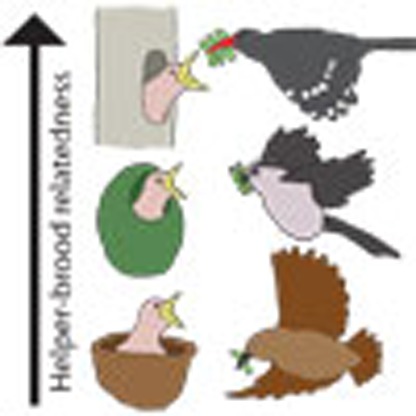

Inclusive fitness theory and the process of kin selection^1^ provide the principal theoretical framework for our understanding of the major evolutionary transitions in sociality in the history of life on earth^2^. Comparative analyses of the transitions to multicellularity by unicellular organisms^3^ and to eusociality in social Hymenoptera^4^,^5^ show that both were associated with high relatedness resulting from clonality and lifetime monogamy, respectively. Likewise, in vertebrate cooperative breeding systems, social groups are usually composed primarily of close relatives^6^,^7^ and interspecific studies show that alloparental care by helpers is more likely to evolve in species with low promiscuity^8^,^9^. Furthermore, many studies have demonstrated a positive effect of helpers on the fitness of kin^10^,^11^,^12^,^13^, although there may also be direct fitness benefits of helping in some species, and exclusively so in those cases where cooperative behaviour occurs only among non-kin^14^.

Hamilton's Rule predicts the conditions under which kin-selected cooperation should evolve, and the broad expectation is that altruistic behaviour is more likely or investment in cooperation should be greater as the relatedness between an actor and recipient increases^1^. Support for this prediction comes from studies showing that potential helpers are more likely to help when recipients are close kin (for example, refs 15, 16, 17) and that, among those individuals that do help, close relatives provide more care than distant relatives in several species (for example, refs 17, 18, 19). Meta-analyses of kin discrimination in cooperatively breeding vertebrates have reported a significant level of kin preference in helping across species^20^,^21^. However, those studies also found that the strength of kin discrimination varied greatly across species, with a substantial proportion of species showing little or no preference for helping kin. Moreover, while kin discrimination was evident in helpers' decisions of whether to help or not, there was no evidence for kin discrimination in the amount of help provided by helpers^21^. Thus, we currently have no explanation for the considerable inter-specific variation in the amount of effort that helpers contribute towards cooperative brood care.

When an individual provides care for young, their investment is ultimately a function of two key evolutionary processes. First, an individual should trade off the fitness gained from current investment against the costs of that investment for future fitness gains^22^,^23^,^24^. Second, an individual's optimal strategy for investment will also depend on the investment of others in the same brood^25^. The outcome of these evolutionary investment games is extremely variable across species, with care being provided uniparentally, biparentally, cooperatively or not at all^26^,^27^. Among cooperative species, in some cases helpers assume most responsibility for brood care, in its most extreme form resulting in the reproductive specialization of many social insects where all care is alloparental. In others, helpers work at a far lower rate than parents in caring for a brood so that most care is parental. Here we use a comparative analysis to test the hypothesis that variation in the contribution of helpers to cooperative brood care among species of cooperatively breeding birds is predicted by inclusive fitness theory. We compared the work rate of helpers across species, predicting that the investment in helping behaviour across species should be consistent with Hamilton's Rule; that is, that, all else being equal, investment should increase with relatedness. We used a measure of helper investment that is comparable across species and ask whether mean species-specific helper investment is positively related to the mean kinship of helpers to the recipients of their alloparental care. Comparing helper effort across species, we find that helper investment increases with their kinship to the brood, thereby demonstrating a key role for kin selection in the evolution of cooperative breeding in birds.

## Results

### Quantifying helper effort

To obtain a standardized measure of helper effort across species, we used parental effort as a benchmark against which we compared the work rate of helpers, under the assumption that parents are closely related to the brood they care for, omitting species where this was known not to be the case (see Methods). Using all the published sources available, we determined the mean provisioning rate of helpers and expressed this as a proportion (helper effort, %) of the provisioning rate of parents. The benchmark parental provisioning rate was weighted according to the sex ratio of helpers, and was adjusted to mean group size and brood size, and standard nestling age within species. Mean kinship of helpers to brood (coefficient of relatedness, *r*) was determined from published pedigree information, supplemented wherever possible by relatedness estimates derived from genetic analyses.

Kinship is not the only potential predictor of helper effort, and so we also investigated the influence of three other variables. First, in cooperative vertebrates where helpers are totipotent and thus have the potential to become breeders in the future, the benefits of current investment as a helper must be traded off against the costs of that investment for future reproductive investment as a breeder. When the probability of future independent breeding is low (that is, in the terminology of Emlen^28^ constraints are high) helper investment should be high, and vice versa. We used the proportion of breeding pairs in study populations that had helpers as a measure of reproductive constraint. Our rationale is that when most individuals in a population are able to breed independently, constraints on reproduction are low and few pairs have helpers, for example, western bluebird *Sialia mexicana*^29^. When constraints on successful independent reproduction are high, most pairs will have helpers, for example, white-winged chough *Corcorax melanorhamphos*^30^.

Second, individuals should take account of the care provided by others when making parental investment decisions^25^, so individual carers should adjust their care according to the number of carers at a nest. In cooperative breeding systems, breeders often benefit from reduced reproductive costs if they reduce their own provisioning rate when helped^31^,^32^. Such adjustments in care in relation to work-force may not be symmetrical. For example, as work-force increases breeders may reduce their effort disproportionately so that the relative effort of helpers increases. The mean group size (breeders+helpers) of each species was determined from published sources, excluding breeders without any helpers and any non-helping group members. It could be argued that such a measure should also take account of the number of recipients (that is, brood size), so in a second analysis we substituted carer/nestling for group size, other variables remaining the same.

Third, the care of helpers could vary consistently in relation to their sex. Therefore, we included average helper sex ratio (proportion of helpers that were male), determined from published sources, as a factor in analyses. We made no explicit prediction about the direction of any effect, although following the logic concerning constraints on reproduction, it could be argued that in avian cooperative breeders, where helpers are typically male, females have a higher probability of dispersing and hence a higher probability of independent reproduction outside of their natal group. Therefore, female helpers might be expected to work less hard than males, although this prediction is more likely to be true within species than across species.

**Figure 1:**
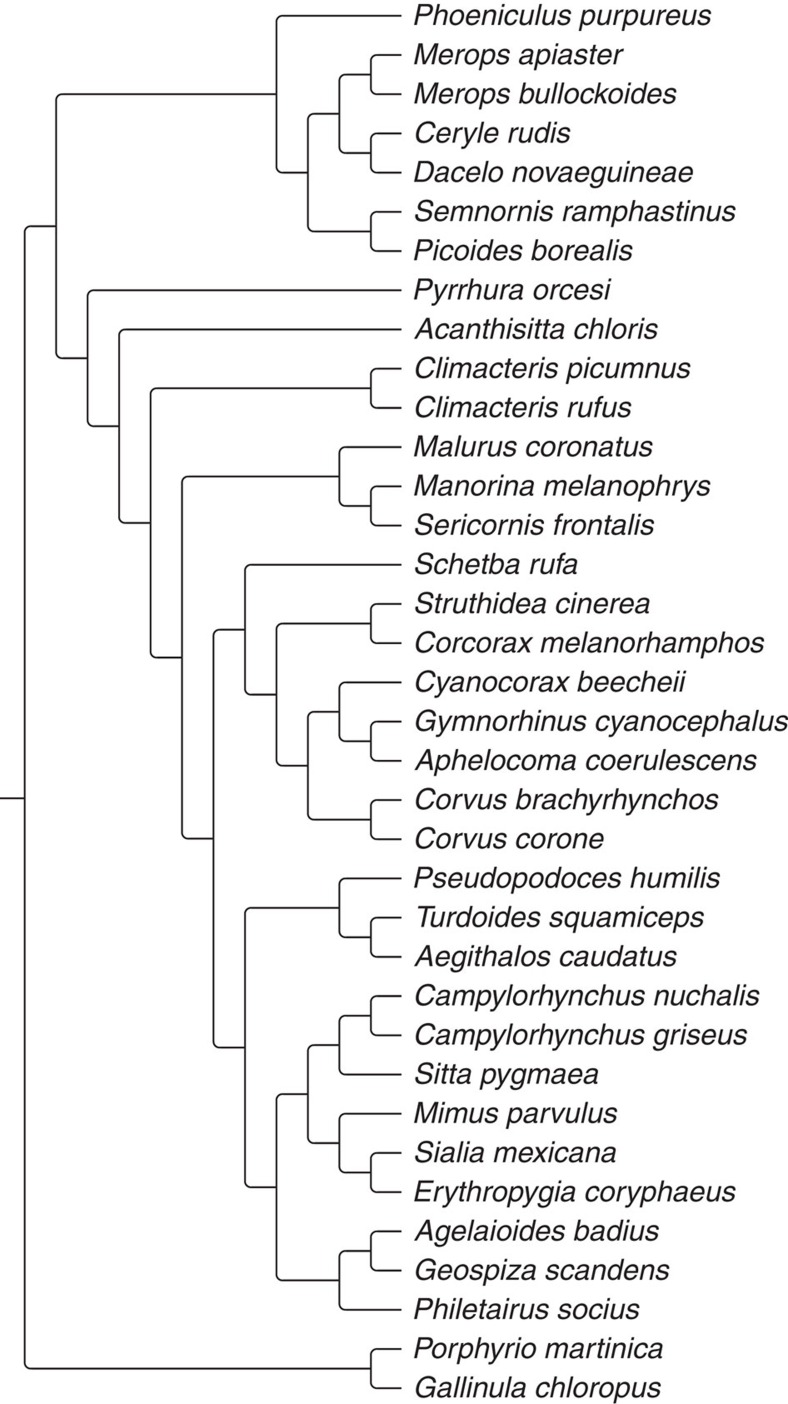
Sample phylogeny of the 36 species used in the PGLS analysis (data from 33).

Data on all key variables were available for a total of 36 species spanning 23 families (Fig. 1; Supplementary Table 1). To test the combined effects of kinship, helper sex ratio, group size and percentage of nests with helpers on helper effort, we used phylogenetic generalized least squares (PGLS) analysis, with phylogenetic information from Jetz *et al*.^33^. An issue for many comparative analyses in the field of behavioural ecology is heterogeneity in the quality of data that arises due to differences such as sample size and study duration^34^,^35^. In our analyses, we used a novel statistical approach to allow for this. We added to our PGLS model a variance term as a random effect that allows for variance components relating to differences in data quality between studies (scored on a qualitative scale). The method allows us to measure the difference in variance across studies, and to then control for this variance heterogeneity in the results (see Methods for details).

### Effect of kinship to brood on helper effort

**Figure 2:**
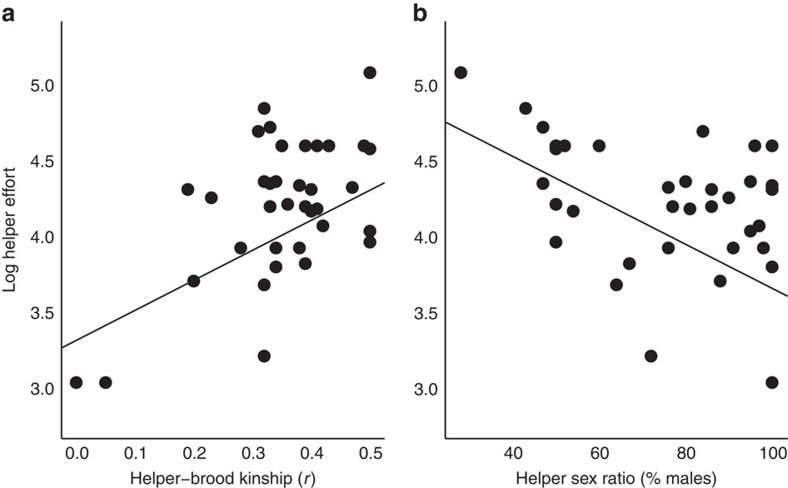
Helper effort varies with helper–brood kinship and helper sex ratio. Across 36 bird species, helper effort (log-transformed) was (**a**) positively related to helper–brood kinship (log (effort)=1.98 × kinship+3.32; *t*=3.40, *P*=0.002, *R*^*2*^=0.23) and (**b**) negatively related to the proportion of male helpers (log(effort)=−0.015 × % males+5.11; *t*=4.83, *P*<0.0001, *R*^*2*^=0.44). Panels show species values and regression lines are fitted by the PGLS models to the full data set. The effect of kinship was still evident when excluding the two outlying species with very low helper–brood kinship (*t*=2.03, *P*=0.05). Results for the other variables included in the PGLS analysis are provided in Table 1, alongside the full results of the lmekin analysis for comparison (see Methods).

Across all species, the amount of care provided by helpers increased with their kinship to the brood, supporting the hypothesis that helper effort is consistent with Hamilton's rule across species (Fig. 2a; Table 1). Helper effort approached 100% (that is, very similar to that of breeders) when *r* was close to 0.5. This is an important finding because it supports the prediction of inclusive fitness theory. Moreover, our approach is not subject to the limitations of meta-analyses of intraspecific kin discrimination that may underestimate the role of kinship in helping decisions. Specifically, intraspecific kin discrimination in helper effort is not an inevitable consequence of kin-selected helping because discrimination should be selected for only when there is a significant risk of caring for non-kin, which may not be the case in species with stable family structures^21^. Furthermore, a significant proportion of helpers may care for non-kin even in a kin-selected cooperative breeding system if the costs of caring are low and the potential indirect benefits are high; under such conditions helpers may be expected to make acceptance errors when assessing kinship^36^.

### Effect of ecological constraints and sex ratio on helper effort

Helper effort was not predicted by the percentage of nests with helpers or group size (Table 1). Substituting carers/nestling for group size did not change the results. However, helper effort was found to decrease significantly with the proportion of helpers that were male (Fig. 2b; Table 1). This effect was not due to greater provisioning effort by females than males within species: in those species where information on provisioning effort was provided for each sex, neither female helpers nor female breeders provisioned at a significantly higher rate than their male counterparts (one-tailed one-sample Wilcoxon test, helpers: *n*=12, *P*=0.69; breeders: *n*=22, *P*=1). Therefore, in species with more female-biased helper sex ratios, helpers of both sexes work harder relative to parental effort. Among cooperatively breeding bird species, helper sex ratios tend to be male-biased as a consequence of female-biased dispersal and male philopatry^37^. More female-biased sex ratios among helpers could thus reflect stronger ecological constraints on dispersal, resulting in females remaining in their natal territory. Lower rates of female dispersal and immigration into other groups are in turn expected to lead to higher average relatedness within groups, which may then select for greater provisioning effort by helpers. Consistent with this hypothesis, more female-biased helper sex ratios were associated with higher ecological constraints (that is, a higher proportion of groups with helpers; PGLS, *t*=−4.87, *P*<0.0001) and also with higher relatedness of helpers to brood (PGLS, *t*=−2.79, *P*=0.009). Increased kinship to brood arising from strong ecological constraints and reduced female dispersal thus provides a potential explanation for the increased provisioning effort of helpers in species with more female-biased helper sex ratios.

## Discussion

We found that the inter-specific variation in cooperative care provided by helpers in avian cooperative breeding systems is consistent with Hamilton's Rule: helper contributions to brood care increases as the relatedness of helpers to recipients increases. This finding provides an explanation for at least some of the previously unexplained variation in helper investment across species. Furthermore, this conclusion supports the findings of previous meta-analyses of intraspecific variation in helper effort that revealed consistent discrimination in favour of kin by helpers^20^,^21^. Of course, this does not necessarily imply that kin-selected gains are the sole fitness benefits derived by helpers in avian cooperative breeding systems, where cooperation may also yield direct fitness benefits, for example, increased survivorship or inheritance of the breeding territory^14^. Overall, however, our findings reaffirm the central role of relatedness in the expression of cooperative behaviours in the light of recent debate^38^,^39^,^40^ and demonstrate the continuing utility of inclusive fitness theory in our understanding of social evolution.

## Methods

### Estimation of helper effort

A critical requirement of the analysis was to develop a measure of helper effort that is comparable across species. The mean rate at which helpers visit the nest to provision nestlings (for example, feeds or biomass delivered per hour, or per nestling per hour) is inappropriate because provisioning rates are subject to species-specific ecological and life-history factors, such as diet, brood-size, mode of development and predation rates, resulting in wide variation in hourly provisioning rates of more than an order of magnitude among the species for which data were available (for example, 0.21 visits per hour for cactus finch *Geospiza scandens* versus 6.8 visits per hour for long-tailed tit *Aegithalos caudatus*; see Supplementary Table 1). Likewise, the proportion of total food or feeds provided by helpers is inappropriate because measures relative to total effort will inevitably depend on the number of carers per brood; for example, two helpers providing food at the same rate in two groups of different size would be regarded as having different provisioning rates. Instead, we take parental provisioning rates as the benchmark against which helper effort was measured.

Published studies were used to determine the provisioning rates of male and female breeders, and of helpers. Because breeders often adjust their own provisioning rate in the presence of helpers^32^, we used provisioning rates of breeders in helped groups as the benchmark, so that provisioning rates of helpers and breeders were obtained from individuals working in the same social environment. Helper effort was expressed as a proportion of breeder effort on a continuous scale, thus helpers who visited the nest at the same rate as breeders scored 100%, those that visited at half the frequency of breeders scored 50%, and so on. Note that helper effort can exceed 100% in cases where helpers provision offspring at a greater rate than breeders. We controlled for several other factors likely to influence provisioning rates. First, we calculated helper effort for the average size of helped broods at a given age. Second, provisioning effort of individual breeders and helpers often varies with the number of helpers at helped nests (for example, refs 41, 42, 43), so we used helper and breeder provisioning rates corrected to the average group size of pairs with helpers. Third, in those species where the provisioning rates of male and female breeders or male and female helpers differed, a species-specific value for helper effort was obtained by multiplying the provisioning rates for mothers and fathers by the proportion of female and male helpers respectively and then summing these values to obtain an overall estimate of provisioning rate for the weighted average parent in each species, against which the contribution of the average helper was then compared. Thus, if 70% of helpers were male, the average effort of helpers was quantified against a benchmark breeder effort that was weighted 70:30 in favour of the male's effort.

Three of the thirty-six species used in the analysis have two distinct categories of helpers: primary and secondary helpers in pied kingfishers *Ceryle rudis*^44^, juvenile and adult helpers in rifleman *Acanthisitta chloris*^45^, and unrelated and related males in white-browed scrubwrens *Sericornis frontalis*^46^. In each case, we used the latter category of helpers because either they extended the range of relatedness in our sample (pied kingfisher), their effort was directly comparable to that of adults (rifleman), or they had no direct parentage of nestlings (white-browed scrubwren). In nearly all species, all group members helped to care for offspring. However, in a few cases (for example, white-browed scrubwren and carrion crow *Corvus corone*), some individuals were never observed feeding broods and these non-helping subordinates were not included when calculating helper effort or group size.

Finally, a key assumption in our approach to testing the kin selection hypothesis is that breeders are themselves closely related to the brood that they are feeding. If this assumption is violated to any great degree then using parental effort as a benchmark against which helper effort is measured becomes meaningless. Therefore, we excluded from our analysis those species with high rates of extra-pair paternity (>20%), such as the brown jay *Cyanocorax morio*^47^ and superb fairy-wren *Malurus cyaneus*^48^, even though detailed information on all other variables was available. This criterion of close breeder relatedness to offspring also necessitated the exclusion of several species with otherwise good data on provisioning effort of helpers because of their complex mating system and hence complicated patterns of parentage. Such species included joint- or plural-breeding cooperative species, including the Seychelles warbler *Acrocephalus sechellensis*^49^, acorn woodpecker *Melanerpes formicivous*^50^ and grey-crowned babbler *Pomatostomus temporalis*^51^. However, one species with a polygamous mating system that fell into this category, the white-browed scrubwren^46^ was included because data were available on relative helper effort in a discrete subset of groups composed of a monogamous pair plus helpers^52^, where we assumed that helper decision rules would be selected for such specific social circumstances. Last, we omitted all cases of cooperative polygamy where all group members are hopeful reproductives rather than helpers in the conventional sense, as in the Galapagos hawk *Buteo galapagoensis*^53^, alpine accentor *Prunella collaris*^54^, dunnock *Prunella modularis*^55^ and Taiwan yuhina *Yuhina brunneiceps*^56^. In these species, there is no parental benchmark where *r*=0.5 against which to measure helper effort.

### Estimation of kinship

The average coefficient of relatedness (*r*) between helpers and the brood they cared for was determined from published sources. In most species (64% of *n*=36), relatedness was estimated from pedigree data alone, where certain proportions of helpers were known to be full-siblings, half-siblings, unrelated and so on to the brood they fed. If the pedigree information needed to estimate relatedness was not reported or was unknown for a substantial proportion of helpers (a particular problem for short-term studies), those species were excluded from the data set. In three cases (8% of *n*=36), where published kinship data were incomplete, estimates of *r* were supplemented using survival rates of helpers and breeders to determine turnover in group membership, and hence the proportion of helpers assisting full-siblings, half-siblings or unrelated young. In a minority of cases (36% of *n*=36) where the genetic relatedness of helpers to broods or breeders had been estimated from genotype data, rather than using computed relatedness coefficients from genetic data alone, we used inferred pedigree relationships (for example, first-order relative, second-order relative and so on) where possible to derive relatedness estimates that were comparable to those derived directly from pedigrees.

### Sex ratio of helpers

All studies that reported the provisioning effort of breeders and helpers, and for which the relatedness of helpers to broods could be estimated, also reported the sex ratio of helpers. Furthermore, sex ratio data were usually available for the same sample of helpers whose provisioning effort was reported. Sex ratio is expressed as the proportion of helpers that were male. In those studies where the provisioning rates of male and female helpers were reported separately and differed proportionally with respect to the effort of male and female breeders, mean helper effort weighted according to helper sex ratio was calculated.

### Proportion of breeding pairs helped

As an indirect measure of constraints on reproductive opportunities, we used the proportion of breeding pairs that were assisted by helpers. This information was reported in all species for which provisioning and relatedness data were available, and usually for the same sample of birds for which provisioning rates and kinship were calculated. The rationale is that in those species, such as the white-winged chough and apostlebird *Struthidea cinerea*, where all breeders have helpers^30^,^42^, the constraints on independent reproduction are severe. In contrast, in those species such as the western bluebird and pinyon jay *Gymnorhinus cyanocepahlus* where a small proportion of pairs are helped^29^,^57^, the constraints on independent reproduction are assumed to be weak because most individuals breed. We hypothesised that, if helping is costly^58^, helper effort should be higher in those instances where the opportunities for future independent reproduction are lower.

### Group size

The size of breeding units (including breeders and helpers) was recorded for those breeding units that had helpers; that is, excluding unassisted breeders. This measure was reported routinely for all species for which provisioning and relatedness data were available and the relevant information was usually available for the same sample of breeding groups for which provisioning data and kinship were available. The rationale for including this as a factor that may potentially influence helper effort is twofold. First, group size may act as an indirect measure of constraints on independent reproduction, following the logic set out above for the proportion of pairs helped. The second reason stems from the load-lightening hypothesis^31^. Individual provisioning effort may change as group size increases for breeders, helpers or both (for example, refs 41, 42, 43). If breeders benefit disproportionately from an increasing number of helpers by decreasing their own investment disproportionately, it would be predicted that helper provisioning effort (measured relative to breeders) would increase as group size increased across species, and vice versa if helpers benefit more than breeders from an increased work-force.

### Statistical analysis

We tested for the effect of helper-brood kinship on (log-transformed) helper effort across the 36 bird species in our data set using PGLS models^59^,^60^,^61^ (for full details see Supplementary Information). Helper sex ratio, group size and percentage of nests with helpers were fitted as additional predictors in the models. We accounted for phylogenetic uncertainty by applying the models to a set of 1,000 equiprobable trees obtained from the avian molecular phylogeny of Jetz *et al*.^33^ and downloaded from birdtree.org using the Hackett All Species backbone (substituting for the Ericson backbone did not affect the results). In our analysis, we were aware that there are considerable methodological differences across studies that affected our confidence in the estimates of the key variables we collated. Such variation can be accommodated within the PGLS framework if a good estimate of variance is available for each observation. Unfortunately, such estimates were not widely available, therefore we assigned each study a qualitative ordinal score of data quality from 1 (weak) to 3 (strong). This score recognizes the fact that some studies were based on detailed behavioural, demographic and genetic data collected on a large sample of individuals over many years, while others were based on field studies lasting only 2–3 years, sometimes with small sample sizes and without genetic confirmation of pedigree relationships. Given these various considerations, it was not possible to define discrete categories of data quality; instead, scores were assigned according to overall impression of data quality. Results of the analyses supported our qualitative assessment of data quality, with studies with low scores contributing the greatest variance and studies with high scores contributing the least variance (Table 1; Supplementary Table 2). Comparison between these results and those obtained from analyses that omitted the data score (Supplementary Table 3) reveals that the sizes of the estimates are similar in both cases but that, as expected, errors around these estimates and associated *P* values are larger when this source of variance is not accounted for.

PGLS models were implemented in R v. 3.2.1 (ref. 62), adapting code previously developed by R.P.F. Owing to the relative complexity of the analysis, we repeated the analyses using the lmekin function in the R package *coxme*^63^. Previous experience suggests that these different methods have their own merits and drawbacks and that it is prudent to fit more complex models using independent implementations where possible. Results from both PGLS and lmekin analyses were in strong agreement (Table 1; Supplementary Table 2).

To permit a meaningful comparison between the provisioning effort of the parents and helpers of a brood, helpers should ideally be adult and have no offspring, either in the helped brood or in another brood, at the time of helping. Five of the thirty-six species in our data set might be considered to violate these rules and we therefore excluded these from a second analysis. These species were (plus reasons for exclusion): moorhen *Gallinula chloropus* (juvenile helpers^64^), rifleman (some helpers fed their own brood simultaneously so effort was shared among nests^65^), bell miner *Manorina melanophrys* (helpers may feed nestlings in multiple synchronous nests^66^), carrion crow and white-browed scrubwren (helper provisioning rules may be influenced by paternity^52^,^67^). We also excluded from this second analysis the white-winged chough *Corcorax melanorhampus*, where provisioning data was available from only a single group^68^, and three species (American crow *Corvus brachyrhynchos*, karoo scrub-robin *Erythropygia coryphaeus* and western bluebird *Sialia mexicana*) with levels of extra-pair paternity of 10–20% extra-pair paternity^69^,^70^,^71^. EPP rates among the remaining species included in the conservative analysis were all <12%. A total of 28 species were thus included in the conservative analysis.

Results of the conservative analysis supported those of the full analysis of all species. Specifically, helper effort was again found to increase significantly with kinship to brood (Supplementary Fig. 1a) and to decrease significantly with the proportion of helpers that were male (Supplementary Fig. 1b), but did not vary with group size or the percentage of nests with helpers. Full results of the conservative analysis are presented in Supplementary Table 2.

Last, to test whether female helpers and breeders contributed more care than their male counterparts, we used one-tailed one-sample Wilcoxon tests to test whether the proportion of total care that was contributed by females in each case was significantly greater than 50%.

### Data availability

The authors declare that the data supporting the findings of this study are available within the article and its Supplementary Information file.

## Additional information

**How to cite this article:** Green, J. P. *et al*. Variation in helper effort among cooperatively breeding bird species is consistent with Hamilton's Rule. *Nat. Commun.* 7:12663 doi: 10.1038/ncomms12663 (2016).

## Supplementary Material

Supplementary Information Supplementary Figure 1, Supplementary Tables 1-3, Supplementary Methods and Supplementary References.

## Figures and Tables

**Table 1:** Effect of kinship to brood, helper sex ratio, group size and the percentage of nests with helpers on helper effort for 36 bird species.

	**PGLS**			**lmekin**		
	**Coefficient±s.e.**	***t***	**P**	**Coefficient±s.e.**	***t***	**P**
Intercept	−0.37±0.38	−0.98	0.33	−0.37±0.37	−0.99	0.33
Kinship to brood	0.48±0.14	3.40	0.002	0.49±0.15	3.39	0.002
Helper sex ratio	−0.66±0.14	−4.83	<0.0001	−0.62±0.15	−4.21	0.0002
Group size	0.07±0.16	0.46	0.65	0.07±0.16	0.46	0.65
% Nests with helpers	−0.15±0.19	−0.82	0.42	−0.13±0.19	−0.65	0.52
						
*Variance components*						
λ	1.00±0.00			1.00±0.00		
Data quality 1	22.33±3.82			26.12±3.06		
Data quality 2	0.11±0.72			0.01±0.29		
Data quality 3	0.18±0.76			2.20±1.83		

Standardized regression coefficients, *t* values and *P* values were obtained from models containing all predictors with helper effort (log-transformed) as the response. Results from PGLS are given alongside those from lmekin analyses for comparison (see Methods). *R*^2^ for all predictors in the full model=0.37 for both analyses. Variance components are scaled to phylogeny (*λ* set to unity). Results indicate that variance associated with low-quality data (score of 1) is greater than that contributed by medium-quality (2) or high-quality (3) data, in line with expectation, and that, in this case, data quality accounts for a substantially greater proportion of variance than phylogenetic similarity.
