# Supplementary material for: Variation in helper effort among cooperatively breeding bird species is consistent with Hamilton's Rule
Source: Nat Commun. 2016 Aug 24;7:12663. doi: 10.1038/ncomms12663 (PMC4999512; doi:10.1038/ncomms12663)
Supplement: Supplementary Information — Supplementary Figure 1, Supplementary Tables 1-3, Supplementary Methods and Supplementary References. [file ncomms12663-s1.pdf]

Supplementary Figures

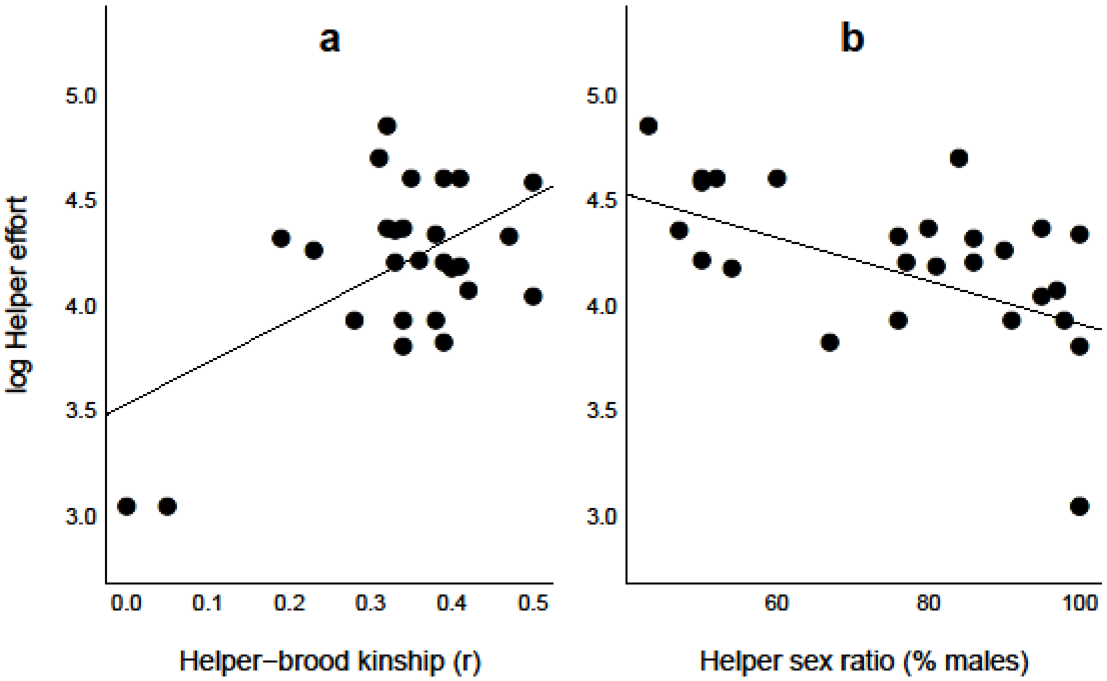

Supplementary Figure 1

Helper effort varies with helper-brood kinship and helper sex ratio. In a conservative analysis of 27 bird species, helper effort (log-transformed) was (a) positively related to helper-brood kinship (log (effort) = 1.98 x kinship + 3.53; t = 2.35, P = 0.03) and (b) negatively related to the proportion of male helpers (log(effort) = -0.01 x % males + 4.94; t = -3.31, P = 0.03). Panels show species values and regression lines are fitted by the PGLS models. Results for the other variables included in the PGLS analysis are provided in Supplementary Table 2, alongside the full results of the lmekin analysis for comparison (see Methods).

Supplementary Tables

Supplementary Table 1. Species data used in comparative analysis of helper effort (expressed as a percentage of parental effort)

Family	Species	Helper effort (%)	Helper-brood kinship (r)	Helper sex ratio (% male)	% nests helped	Group size (n)	Brood size (n)	Data score	References
Acanthisittidae	rifleman[*] *Acanthisitta chloris*	25	0.32	72	13	3.1	3.1	3	1, 2
Aegithalidae	long-tailed tit *Aegithalos caudatus*	75	0.19	86	54	4.0	8.8	3	3-5
Alcedinidae	pied kingfisher[‡] *Ceryle rudis*	21	0.05	100	65	3.6	4.5	3	6
Alcedinidae	laughing kookaburra *Dacelo novaeguineae*	76	0.47	76	66	4.2	2.1	3	7, 8
Certhiidae	bicolored wren *Campylorhynchus griseus*	66	0.41	81	30	3.5	2.8	2	9-11
Certhiidae	stripe-backed wren *Campylorhynchus nuchalis*	100	0.35	52	67	4.4	2.5	2	12-14
Climacteridae	brown treecreeper[‡] *Climacteris picumnus*	59	0.42	97	38	3.6	2.3	3	15, 16
Climacteridae	rufous treecreeper[§] *Climacteris rufa*	67	0.33	77	59	3.7	1.3	2	17
Corcoracidae	white-winged chough[*] *Corcorax melanorhamphos*	113	0.33	47	100	6.8	2	1	18-21
Corcoracidae	Apostlebird *Struthidea cinerea*	100	0.39	50	100	7.6	2.7	3	22-24
Corvidae	Florida scrub jay *Aphelocoma coerulescens*	65	0.40	54	55	3.7	2.4	3	25-27
Corvidae	American crow[*] *Corvus brachyrhynchos*	162	0.50	28	25	3.4	1.9	1	28-30
Corvidae	carrion crow[*] *Corvus corone*	40	0.32	62	75	3.6	2.5	3	31-35
Corvidae	purplish-backed jay *Cyanocorax beechei*	68	0.36	50	81	4.2	2.3	2	36, 37
Corvidae	pinyon jay *Gymnorhinus cyanocephalus*	57	0.50	95	11	3.0	1.2	1	38, 39
Corvidae	rufous vanga *Schetba rufa*	51	0.28	98	37	3.1	2.1	2	40, 41
Fringillidae	bay-winged cowbird *Agelaioides badius*	71	0.23	90	95	3.5	4	1	42
Maluridae	purple-crowned fairy-wren	51	0.38	76	48	4.6	2.9	3	43-45; S. A. Kingma, pers.

	Malurus coronatus								comm.
Meliphagidae	bell miner[*]<br>*Manorina melanophrys*	41	0.20	88	100	10.6	1.9	3	46-48
Meropidae	European bee-eater<br>*Merops apiaster*	51	0.34	91	19	3.3	5.3	3	49
Meropidae	white-fronted bee-eater<br>*Merops bullockoides*	78	0.33	47	50	3.5	1.8	3	50, 51
Muscicapidae	Karoo scrub-robin[*]<br>*Erythropygia coryphaeus*	75	0.40	100	15	3.2	2.5	3	52
Muscicapidae	western bluebird[*,‖]<br>*Sialia mexicana*	100	0.43	96	7	3.0	4.7	3	53
Pardalotidae	white-browed scrubwren[*,¶]<br>*Sericornis frontalis*	100	0.49	100	54	3.3	2.3	2	54-57
Paridae	ground tit<br>*Pseudopodeces humilis*	77	0.38	100	39	3.1	5.5	3	58
Passeridae	cactus finch<br>*Geospiza scandens*	21	0	100	100	3.4	1.6	1	59
Passeridae	sociable weaver<br>*Philetairus socius*	79	0.34	80	56	4.0	3	3	60-62
Phoeniculidae	green woodhoopoe<br>*Phoeniculus purpureus*	128	0.32	43	91	5.1	1.6	3	63-65
Picidae	red-cockaded woodpecker<br>*Picoides borealis*	79	0.32	95	40	3.2	2.1	3	66-70
Psitticidae	El Oro parakeet[◊]<br>*Pyrrhura orcesi*	46	0.39	67	74	4.3	4	2	71, 72
Rallidae	Moorhen[*]<br>*Gallinula chloropus*	53	0.50	50	67	3.5	2.4	2	73, 74
Rallidae	purple gallinule[‖]<br>*Porphyrio martinica*	98	0.50	50	63	4.2	2.3	2	75, 76
Ramphastidae	toucan barbet<br>*Semnornis ramphastinus*	67	0.39	86	57	3.3	1.1	1	77
Sittidae	pygmy nuthatch<br>*Sitta pygmaea*	45	0.34	100	30	3.4	5.2	2	78, 79
Sturnidae	Galapagos mockingbird<br>*Mimus parvulus*	110	0.31	84	34	3.2	2.5	2	80-82
Sylviidae	Arabian babbler<br>*Turdoides squamiceps*	100	0.41	60	100	4.8	3.1	3	83-87

[*]Species omitted from conservative analysis; [†]Data for secondary helpers only; [‡]Data for groups, not super-groups; [§]Data for resident helpers only; [‖]Data for adult helpers only; [¶]Data for groups where helpers are unrelated to breeding female; [◊]Data for groups where helpers are unrelated to breeding female. For further explanation of footnotes, see Methods. Details of the data used to estimate parameters are available from the authors upon request.

Supplementary Table 2: Effect of kinship to brood, helper sex ratio, group size and the percentage of nests with helpers on helper effort from the conservative analysis of 27 bird species.

	PGLS			lmekin		
	**Coefficient ± SE**	***t***	***P***	**Coefficient ± SE**	***t***	***P***
Intercept	-0.14 ± 0.36	-0.39	0.70	-0.14 ± 0.36	-0.39	0.70
Kinship to brood	0.37 ± 0.16	2.35	0.03	0.37 ± 0.16	2.36	0.03
Helper sex ratio	-0.58 ± 0.18	-3.31	0.003	-0.58 ± 0.18	-3.32	0.003
Group size	0.10 ± 0.18	0.56	0.58	0.10 ± 0.18	0.55	0.59
% nests with helpers	-0.11 ± 0.25	-0.45	0.66	-0.11 ± 0.25	-0.45	0.66
**Variance components**						
ʎ	1.00 ± 0.00			1.00 ± 0.00		
Data Quality 1	45.15 ± 5.94			40.85 ± 5.00		
Data Quality 2	0.35 ± 1.85			0.10 ± 0.77		
Data Quality 3	0.00 ± 0.00			0.00 ± 0.00		

Standardised regression coefficients, *t* values and *P* values were obtained from models containing all predictors with helper effort (log-transformed) as the response. Results from PGLS are given alongside those from lmekin analyses for comparison (see Methods). R^2 for all predictors in the full model = 0.50 for both analyses. Variance components are scaled to phylogeny (ʎ set to unity). Results indicate that variance associated with low-quality data (score of 1) is greater than that contributed by medium-quality (2) or high-quality (3) data, in line with expectation, and that, in this case, data quality accounts for a substantially greater proportion of variance than phylogenetic similarity.

Supplementary Table 3: Results of the PGLS analysis of 36 bird species when data quality is not accounted for.

	**Coefficient ± SE**	***t***	***P***
Intercept	-0.15 ± 0.37	0.39	0.70
Kinship to brood	0.55 ± 0.16	3.49	0.002
Helper sex ratio	-0.51 ± 0.18	2.87	0.02
Group size	-0.00 ± 0.18	0.15	0.89
% nests with helpers	0.04 ± 0.23	0.16	0.87

Standardised regression coefficients, *t* values and *P* values were obtained from a model containing all predictors with helper effort (log-transformed) as the response.

Supplementary Methods

Details of the PGLS methodology used in the analyses of helper effort. Phylogenetic Generalized Least Squares (PGLS) accounts for the non-independence that results from shared evolutionary history. The basis for the approach is a linear model in which a vector of *n* observations, **y**, is modelled as a function of an *n* x *k* matrix of *k* predictors **X**[88]:

$$\mathbf{y} = \mathbf{Xb} + \mathbf{e} \qquad (1)$$

In order to account for phylogenetic non-independence, the errors **e** are assumed to follow a multivariate normal distribution, the covariance matrix of which accounts for the expected similarity resulting from non-independence. The basic model for this expected covariance is:

$$\mathbf{W} = \lambda\mathbf{V} + (1-\lambda)\mathbf{H} \qquad (2)$$

V is an *n x n* matrix of shared phylogenetic path distances that are shared among species. The off-diagonal entries of **V** measure expected covariances owing to phylogenetic similarity, whilst diagonal entries measure the expected variance among species. λ modifies the model to account for additional non-phylogenetic dependence, for example resulting from species-specific evolution or measurement error[89,90]. A proportion (1 - λ) of the expected variance is non-phylogenetic and this is added to the diagonal of scaled **V**. **H** is a diagonal matrix, the entries of which are the diagonal elements of **V**. **W** is thus a composite of phylogenetic and non-phylogenetic sources of residual variation.

Equation (1) may be fitted to data by treating λ in equation (2) as an unknown parameter, then using maximum likelihood to estimate this parameter along with the parameters of equation (1). This approach, of estimating the variance structure as part of the model, is known as Estimated or Feasible Generalized Least Squares.

To account for variation in data quality among the studies used in our analyses, we assigned each study a qualitative ordinal score of data quality from 1 (weak) to 3 (strong) (see Methods). Data quality scores were incorporated into the analysis by further modifying equation (2):

$$\mathbf{W} = \lambda\mathbf{V} + (1-\lambda)\mathbf{I} + \varepsilon_H^2\mathbf{E}_H + \varepsilon_M^2\mathbf{E}_M + \varepsilon_L^2\mathbf{E}_L \qquad (3)$$

$\mathbf{E_H}$, $\mathbf{E_m}$ and $\mathbf{E_L}$ are matrices coding for the quality of each study (1 indicates that the study is of the respective quality, 0 indicates not). The variance components ε_H, ε_M and ε_L measure the additional variance attributable to each category. These were estimated by maximum likelihood along with λ and the best-fit parameters of equation (1). Estimates of ε_H, ε_M and ε_L from the analyses supported the qualitative assessment of data quality, with low quality studies contributing the greatest variance and high quality studies contributing the least variance (Table 1; Supplementary Table 2).

The above assumes that we have one phylogeny. In our analyses, we accounted for phylogenetic uncertainty by applying the models to a set of 1000 equiprobable trees (see Methods). An analysis on tree *i* yields a parameter estimate b_{ij} for parameter *j*, together with associated variance s_{ij}^2. Across *t* trees, the mean and variance of the parameter value is, accounting for both statistical and phylogenetic variance:

$$\hat{b}_j = \frac{1}{t}\sum_{i=1}^{t} b_{ij}$$

and:

$$\hat{s}_j^2 = var(s_{ij}^2) + \frac{1}{t}\sum_{i=1}^{t} s_{ij}^2$$

Where $var(s_{ij}^2)$ is the variance of estimates across all of the trees.

Supplementary References

1. Preston, S. A. J., Briskie, J. V., Burke, T. & Hatchwell, B. J. Genetic analysis reveals diverse kin-directed routes to helping in the rifleman *Acanthisitta chloris*. *Mol. Ecol.* **22**, 5027-5039 (2013)
2. Preston, S. A. J. Alternative routes to cooperative breeding in the rifleman, *Acanthisitta chloris*. PhD Thesis, University of Sheffield (2012)
3. MacColl, A. D. C. & Hatchwell, B. J. Sharing of caring: nestling provisioning behaviour of long-tailed tit, *Aegithalos caudatus*, parents and helpers. *Anim. Behav*. **66**, 955-964 (2003)
4. Nam, K. B., Simeoni, M., Sharp, S. P. & Hatchwell, B. J. Kinship affects investment by helpers in a cooperatively breeding bird. *P. Roy. Soc. Lond. B* **277**, 3299-3306 (2010)
5. Hatchwell, B. J., Gullett, P. R. & Adams, M. J. Helping in cooperatively breeding long-tailed tits: a test of Hamilton's rule. *Philos. T. Roy. Soc. B* **369**, 20130565 (2014).
6. Reyer, H. U. in *Cooperative breeding in birds: long-term studies of ecology and behavior* (ed. Stacey, P. B. & Koenig, W. D.) 529-557 (Cambridge University Press, 1990)
7. Legge, S. Helper contributions in the cooperatively breeding laughing kookaburra: feeding young is no laughing matter. *Anim. Behav*. **59**, 1009-1018 (2000)
8. Legge, S & Cockburn, A. Social and mating systems of cooperatively breeding laughing kookaburras (*Dacelo novaeguineae*). *Behav. Ecol. Sociobiol*. **47**, 220-229 (2000)
9. Austad, S. N. & Rabenold, K. N. Reproductive enhancement by helpers and an experimental inquiry into its mechanism in the bicolored wren. *Behav. Ecol. Sociobiol*. **17**, 19-27 (1985)
10. Austad, S. N. & Rabenold, K. N. Demography and the evolution of cooperative breeding in the bicolored wren, *Campylorhynchus griseus*. *Behaviour* **97**, 308-324 (1986)
11. Haydock, J., Parker, P. G. & Rabenold, K. N. Extra-pair paternity uncommon in the cooperatively breeding bicolored wren. *Behav. Ecol. Sociobiol*. **38**, 1-16 (1996)
12. Rabenold, K. N. Cooperative enhancement of reproductive success in tropical wren societies. *Ecology* **65**, 871-885 (1984)
13. Rabenold, K. N. Cooperation in breeding by non-reproductive wrens: kinship, reciprocity and demography. *Behav. Ecol. Sociobiol*. **17**, 1-17 (1985)
14. Rabenold, K. N. in *Cooperative breeding in birds: long-term studies of ecology and behavior* (ed. Stacey, P. B. & Koenig, W. D.) 159-196 (Cambridge University Press, 1990)
15. Doerr, E. D. & Doerr, V. A. J. Comparative demography of treecreepers: evaluating hypotheses for the evolution and maintenance of cooperative breeding. *Anim. Behav*. **72**, 147-159 (2006)
16. Doerr, E. D. & Doerr, V. A. J. Positive effects of helpers on reproductive success in the brown treecreeper and the general importance of future benefits. *J Anim. Ecol*. **76**, 966-976 (2007)
17. Luck, G. W. The demography and cooperative breeding behaviour of the rufous treecreeper, *Climacteris rufa*. *Aust. J. Zool*. **49**, 515-537 (2001)
18. Heinsohn, R. Cooperative enhancement of reproductive success in white-winged choughs. *Evol. Ecol*. **6**, 97-114 (1992)
19. Rowley, I. Communal activities among white-winged choughs *Corcorax melanorhamphus*. *Ibis* **120**, 178-197 (1978)
20. Beck, N. R., Peakall, R. & Heinsohn, R. Social constraint and an absence of sex-biased dispersal drive fine-scale genetic structure in white-winged choughs. *Mol. Ecol*. **17**, 4346-4358 (2008)

21. Boland, C. R. J., Heinsohn, R. & Cockburn, A. Deception by helpers in cooperatively breeding white-winged choughs and its experimental manipulation. *Behav. Ecol. Sociobiol*. **41**, 251-256 (1997)
22. Woxvold, I. A. & Magrath, M. J. L. Helping enhances multiple components of reproductive success in the cooperatively breeding apostlebird. *J. Anim. Ecol*. **74**, 1039-1050 (2005)
23. Woxvold, I. A., Adcock, G. J. & Mulder, R. A. Fine-scale genetic structure and dispersal in cooperatively breeding apostlebirds. *Mol. Ecol*. **15**, 3139-3146 (2006)
24. Woxvold, I. A., Mulder, R. A. & Magrath, M. J. L. Contributions to care vary with age, sex, breeding status and group size in the cooperatively breeding apostlebird. *Anim. Behav*. **72**, 63-73 (2006)
25. McGowan, K. J. & Woolfenden, G. E. Contributions to fledgling feeding in the Florida scrub jay. *J. Anim. Ecol*. **59**, 691-707 (1990)
26. Woolfenden, G. E. & Fitzpatrick, J. W. in *Cooperative breeding in birds: long-term studies of ecology and behavior* (ed. Stacey, P. B. & Koenig, W. D.) 239-266 (Cambridge University Press, 1990)
27. Mumme, R. L. Do helpers increase reproductive success? An experimental analysis in the Florida scrub jay. *Behav. Ecol. Sociobiol*. **31**, 319-328 (1992)
28. Caffrey, C. Female-biased delayed dispersal and helping in American crows. *Auk* **109**, 609-619 (1992)
29. Caffrey, C. Feeding rates and individual contributions to feeding at nests in cooperatively breeding western American crows. *Auk* **116**, 836-841 (1999)
30. Caffrey, C. Correlates of reproductive success in cooperatively breeding western American crows: if helpers help it's not by much. *Condor* **102**, 333-341 (2000)
31. Baglione, V., Marcos, J. M., Canestrari, D. & Ekman, J. Direct fitness benefits of group living in a complex cooperative society of carrion crows, *Corvus corone corone*. *Anim. Behav*. **64**, 887-893 (2002)
32. Baglione, V., Marcos, J. M., Canestrari, D. Cooperatively breeding groups of carrion crow (*Corvus corone corone*) in Northern Spain. *Auk* **119**, 790-799 (2002)
33. Canestrari, D., Marcos, J. M. & Baglione, V. Effect of parentage and relatedness on the individual contribution to cooperative chick care in carrion crows. *Behav. Ecol. Sociobiol*. **52**, 42-428 (2005)
34. Canestrari, D., Marcos, J. M. & Baglione, V. Costs of chick provisioning in cooperatively breeding crows: an experimental study. *Anim. Behav*. **73**, 349-357 (2007)
35. Canestrari, D., Marcos, J. M. & Baglione, V. Helpers but not breeders adjust provisioning effort to year-round territory resource availability in carrion crows. *Anim. Behav*. **76**, 943-949 (2008)
36. Raitt, R. J. & Hardy, J. W. Social behavior, habitat and food of the beechey jay. *Wilson Bull*. **91**, 1-15 (1979)
37. Raitt, R. J., Winterstein, S. R. & Hardy, J. W. Structure and dynamics of communal groups in the beechey jay. *Wilson Bull*. **96**, 206-227 (1984)
38. Marzluff, J. M. & Balda, R. P. in *Cooperative breeding in birds: long-term studies of ecology and behavior* (ed. Stacey, P. B. & Koenig, W. D.) 199-237 (Cambridge University Press, 1990).
39. Marzluff, J. M. & Balda, R. P. *The pinyon jay: behavioural ecology of a colonial and cooperative corvid* (Poyser, 1992)
40. Yamagishi, S., Urano, E. & Eguchi, K. Group composition and contributions to breeding by rufous vangas *Schetba rufa* in Madagascar. *Ibis* **137**, 157-161 (1995)

41. Eguchi, K., Yamagishi, S., Asai, S., Nagata, H. & Hino, T. Helping does not enhance reproductive success of cooperatively breeding rufous vanga in Madagascar. *J. Anim. Ecol*. **71**, 123-130 (2002)
42. Fraga, R. M. The social system of a communal breeder, the bay-winged cowbird *Molothrus badius*. *Ethology* **89**, 195-210 (1991)
43. Kingma, S. A., Hall, M. L., Segelbacher, G. & Peters, A. Radical loss of an extreme extra-pair mating system. *BMC Ecol*. **9**, 15 (2009)
44. Kingma, S. A. Hall, M. L., Arriero, E. & Peters, A. Multiple benefits of cooperative breeding in purple-crowned fairy-wrens: a consequence of fidelity? *J. Anim. Ecol*. **79**, 757-768 (2010)
45. Kingma, S. A., Hall, M. L. & Peters, A. Multiple benefits drive helping behaviour in a cooperatively breeding bird: an integrated analysis. *Am. Nat*. **177**, 486-495 (2011)
46. Clarke, M. F. *et al*., Male-biased sex ratios in broods of the cooperatively breeding bell miner *Manorina melanophrys*. *J. Avian Biol*. **33**, 71-76 (2002)
47. te Marvelde, L., McDonald, P. G., Kzem, A. J. N. & Wright, J. Do helpers really help? Provisioning biomass and prey type effects on nestling growth in the cooperative bell miner. *Anim. Behav*. **77**, 727-735 (2009)
48. Wright, J. McDonald, P. G., te Marvelde, L. Kazem, A. J. N. & Bishop, C. M. Helper effort increases with relatedness in bell miners, but 'unrelated' helpers of both sexes still provide substantial care. *P. Roy. Soc. Lond. B*, rspb20091360 (2009)
49. Lessells, C. M. in *Population biology of passerine birds: an integrated approach* (ed. Blondel, J., Gosler, A., Lebreton, J. D. & McCleery, R.) 357-368 (Springer, 1990)
50. Emlen, S. T. in *Cooperative breeding in birds: long-term studies of ecology and behavior* (ed. Stacey, P. B. & Koenig, W. D.) 487-526 (Cambridge University Press, 1990)
51. Emlen, S. T. & Wrege, P. H. Breeding biology of white-fronted bee-eaters at Nakuru: the influence of helpers on breeder fitness. *J. Anim. Ecol*. **60**, 309-326 (1991)
52. Lloyd, P., Taylor, W. A., du Plessis, M. A. & Martin, T. E. Females increase reproductive investment in response to helper-mediated improvements in allo-feeding, nest survival, nestling provisioning and post-fledgling survival in the Karoo scrub-robin *Cercotrichas coryphaeus*. *J. Avian Biol*. **40**, 400-411 (2009)
53. Dickinson, J. L., Koenig, W. D. & Pitelka, F. A. Fitness consequences of helping behavior in the western bluebird. *Behav. Ecol*. **7**, 168-177 (1996)
54. Magrath, R. D. & Whittingham, L. A. Subordinate males are more likely to help if unrelated to the breeding female in cooperatively breeding white-browed scrubwrens. *Behav. Ecol. Sociobiol*. **41**, 185-192 (1997)
55. Whittingham, L. A. & Dunn, P. O. Male parental effort and paternity in a variable mating system. *Anim. Behav*. **55**, 629-640 (1998)
56. Whittingham, L. A., Dunn, P. O. & Magrath, R. D. Relatedness, polyandry and extra-group paternity in the cooperatively-breeding white-browed scrubwren. *Behav. Ecol. Sociobiol*. **40**, 261-270 (1997)
57. Magrath, R. D. *et al*., Life in the slow lane: reproductive life history of the white-browed scrubwren, an Australian endemic. *Auk* **117**, 479-489 (2000)
58. Lu, X., Yu, L. & Ke, D. Helped ground tit parents in poor foraging environments reduce provisioning effort despite nestling starvation. *Anim. Behav*. **82**, 861-867 (2011)
59. Price, T., Millington, S., Grant, P. R. Helping at the nest in Darwin's finches as misdirected parental care. *Auk* **100**, 192-194 (1983)
60. Covas, R., du Plessis, M. A. & Doutretant, C. Helpers in colonial cooperatively breeding sociable weavers *Philetairus socius* contribute to buffer the effects of adverse breeding conditions. *Behav. Ecol. Sociobiol*. **63**, 103-112 (2008)

61. Covas, R. Dalecky, A., Caizergues, A. & Doutrelant, C. Kin associations and direct vs indirect fitness benefits in colonial cooperatively breeding sociable weavers *Philetairus socius*. *Behav. Ecol. Sociobiol*. **60**, 323-331 (2006)
62. Doutrelant, C. & Covas, R. Helping has signalling characteristics in a cooperatively breeding bird. *Anim. Behav*. **74**, 739-747 (2007)
63. du Plessis, M. A. The role of helpers in feeding chicks in cooperatively-breeding green (red-billed) woodhoopoes. *Behav. Ecol. Sociobiol*. **28**, 291-295 (1991)
64. Ligon, J. D. & Ligon, S. H. The communal social system of the green woodhoopoe in Kenya. *Living Bird* **17**, 159-197 (1978)
65. J. D. Ligon, S. H. Ligon, in *Cooperative breeding in birds: long-term studies of ecology and behavior* (ed. Stacey, P. B. & Koenig, W. D.) 31-65 (Cambridge University Press, 1990)
66. Lennartz, M. R. & Harlow, R. F. The role of parent and helper red-cockaded woodpeckers at the nest. *Wilson Bull*. **91**, 31-335 (1979)
67. Lennartz, M. R., Hooper, R. G. & Harlow, R. F. Sociality and cooperative breeding of red-cockaded woodpeckers, *Picoides borealis*. *Behav. Ecol. Sociobiol*. **20**, 77-88 (1987)
68. Walters, J. R. in *Cooperative breeding in birds: long-term studies of ecology and behavior* (ed. Stacey, P. B. & Koenig, W. D.) 69-101 (Cambridge University Press, 1990)
69. Haig, S. M., Walters, J. R. & Plissner, J. H. Genetic evidence for monogamy in the cooperatively breeding red-cockaded woodpecker. *Behav. Ecol. Sociobiol*. **34**, 295-303 (1994)
70. Khan, M. Z. & Walters, J. R. Effects of helpers on breeder survival in the red-cockaded woodpecker (*Picoides borealis*). *Behav. Ecol. Sociobiol*. **51**, 336-344 (2002)
71. Klauke, N., Segelbacher, G. & Schaefer, H. M. Reproductive success depends on the quality of helpers in the endangered, cooperative El Oro parakeet (*Pyrrhura orcesi*). *Mol. Ecol*. **22**, 2011-2027 (2013)
72. Klauke, N., Jansen, J., Kramer, J. & Schaefer, H. M. Food allocation rules vary with age and experience in a cooperatively breeding parrot. *Behav. Ecol. Sociobiol*. **68**, 1037-1047 (2014)
73. Eden, S. F. When do helpers help? Food availability and helping in the moorhen, *Gallinula chloropus*. *Behav. Ecol. Sociobiol*. **21**, 191-195 (1987)
74. Gibbons, D. W. Juvenile helping in the moorhen, *Gallinula chloropus*. *Anim. Behav*. **35**, 170-181 (1987)
75. Hunter, L. A. The effects of helpers in cooperatively breeding purple gallinules. *Behav. Ecol. Sociobiol*. **18**, 147-153 (1985)
76. Hunter, L. A. Cooperative breeding in purple gallinules: the role of helpers in feeding chicks. *Behav. Ecol. Sociobiol*. **20**, 171-177 (1987)
77. Restrepo, C. & Mondragon, M. L. Cooperative breeding in the frugivorous toucan barbet. *Auk* **115**, 4-15 (1998)
78. Sydeman, W. J. Effects of helpers on nestling care and breeder survival in pygmy nuthatches. *Condor* **91**, 147-155 (1989)
79. Sydeman, W. J., Guntert, M. & Balda, R. P. Annual reproductive yield in the cooperative pygmy nuthatch (*Sitta pygmaea*). *Auk* **105**, 70-77 (1988)
80. Kinnaird, M. F. & Grant, P. R. Cooperative breeding by the Galapagos mockingbird, *Nesomimus parvulus*. *Behav. Ecol. Sociobiol*. **10**, 65-73 (1982)
81. Curry, R. L. Influence of kinship on helping behavior in Galapagos mockingbirds. *Behav. Ecol. Sociobiol*. **22**, 141-152 (1988)
82. Curry, R. L. & Grant, P. R. in *Cooperative breeding in birds: long-term studies of ecology and behavior* (ed. Stacey, P. B. & Koenig, W. D.) 289-332 (Cambridge University Press, 1990)

83. Wright, J. Helping-at-the-nest in Arabian babblers: signalling social status or sensible investment in chicks? *Anim. Behav*. **54**, 1439-1448 (1997)
84. Wright, J. Helpers-at-the-nest have the same provisioning rules as parents: experimental evidence from playbacks of chick begging. *Behav. Ecol. Sociobiol*. **42**, 423-429 (1998)
85. Wright, J. & Dingemanse, N. J. Parents and helpers compensate for experimental changes in the provisioning effort of others in the Arabian babbler. *Anim. Behav*. **58**, 345-350 (1999)
86. Wright, J., Parker, P. G. & Lundy, K. J. Relatedness and chick-feeding effort in the cooperatively breeding Arabian babbler. *Anim. Behav*. **58**, 779-785 (1999)
87. Ridley, A. R. Factors affecting offspring survival and development in a cooperative bird: social, maternal and environmental effects. *J. Anim. Ecol*. **76**, 750-760 (2007)
88. Martins, E. P. & Hansen, T. F. Phylogenies and the comparative method: A general approach to incorporating phylogenetic information into the analysis of interspecific data. *Am. Nat*. **149**, 646-667 (1997)
89. Pagel, M. Inferring evolutionary processes from phylogenies. *Zool. Scr*. **26**, 331-348 (1997)
90. Freckleton, R. P., Harvey, P. H. & Pagel, M. Phylogenetic analysis and ecological data: a review of the evidence. *Am. Nat*. **160**, 712-726 (2002)
